# Parecoxib prevents complications in hepatocellular carcinoma patients receiving hepatic transarterial chemoembolization: a prospective score-matched cohort study

**DOI:** 10.18632/oncotarget.8560

**Published:** 2016-04-02

**Authors:** Zhong-Guo Zhou, Jin-Bin Chen, Hai-Bo Qiu, Ruo-Jing Wang, Jian-Cong Chen, Li Xu, Min-Shan Chen, Yao-Jun Zhang

**Affiliations:** ^1^ Sun Yat-Sen University Cancer Center, State Key Laboratory of Southern China, Collaborative Innovation Center for Cancer Medicine, Guangzhou 510060, P.R. China; ^2^ First Affiliated Hospital of Sun Yat-Sen University, Guangzhou 510060, P.R. China

**Keywords:** parecoxib, hepatocellular carcinoma, transarterial chemoembolization

## Abstract

Transarterial chemoembolization(TACE) is the palliative treatment of choice for patients with unresectable hepatocellular carcinoma (HCC). The 242 patients prospectively enrolled in this study were diagnosed with HCC and received TACE at Sun Yat-Sen University Cancer Center between October 2014 and March 2015. Patients were divided into study and control groups based on whether parecoxib sodium was administered postoperatively. Postoperative pain, body temperature, vomiting, changes in liver function, physical activity level, length of hospital stay, and tumor control were evaluated. Compared to the control group after propensity score matching, the study group presented less severe postoperative fever. The daily maximum temperatures in the study and control groups were 37.39 vs. 37.82°C on postoperative day 1 (*P* < 0.001), 37.10 vs. 37.51°C on day 2 (*P* < 0.001), and 36.90 vs. 37.41°C on day 3 (*P* < 0.001). The study group also exhibited greater physical activity (*P* < 0.05) and had shorter hospital stays (7.21 days vs. 7.92 days, *P* = 0.041). There were no differences in pain scores. Thus administration of parecoxib sodium to HCC patients after TACE effectively relieved fever, promoted postoperative recovery, and shortened the hospital stay.

## INTRODUCTION

Liver cancer ranks as the fifth most common malignancy in males and seventh most common in females, and is the third most common cause of cancer death worldwide [1]. In 2010, there were 358,840 new cases of liver cancer in China and 312,432 deaths from the disease [2, 3]. Due to the occult nature of liver cancers, approximately 80% of liver cancer patients are not candidates for radical resection upon diagnosis. Transarterial chemoembolization (TACE) is the primary palliative treatment of choice for patients with unresectable hepatocellular carcinoma (HCC). A large number of clinical studies have confirmed that TACE prolongs survival [4], and for certain early liver cancers, TACE can be curative [5]. However, TACE is often accompanied by postoperative complications, which include fever, pain, vomiting and liver dysfunction. The appropriate prevention and treatment of such complications are important measures that promote patient recovery.

Parecoxib sodium is the inactive, water-soluble precursor of valdecoxib, which exerts antipyretic, analgesic, and anti-inflammatory effects by selectively inhibiting cyclo-oxygenase-2 (COX-2). This inhibition of COX-2 blocks the conversion of arachidonic acid into prostaglandins. Several clinical studies have shown that parecoxib sodium has a good adjuvant analgesic effect on postoperative pain after nasal endoscopy, orthopedic surgeries, and prostate and gastrointestinal surgeries [6–9]. It was also demonstrated that parecoxib sodium relieves fever and inflammatory responses. In the present study we conducted a prospective non-randomized controlled investigation of the ability of parecoxib sodium to prevent postoperative fever, pain, abnormal liver function, and other adverse reactions after TACE.

## RESULTS

### Study population and baseline clinical characteristics

A total of 242 patients met the inclusion criteria and were enrolled in the study and divided into two groups. Comparison of the clinicopathological characteristics of the two groups showed that they were similar with respect to all parameters analyzed (Table 1).

**Table 1:** Baseline characteristics of all enrolled patients

Characteristics	Total (*n* = 242)	Parecoxib (*n* = 86)	Non-Parecoxib (*n* = 156)	*P*-Value
**Sex (Males:Females)**	202:40	72:14	130:26	0.0917
**Age (years)**	52.8 ± 13.15	52.79 ± 13.45	52.88 ± 11.15	0.4735
**WBC (×10^9^/L)**	6.56 ± 2.12	6.38 ± 1.85	6.82 ± 2.25	0.2687
**HBG (g/L)**	137.23 ± 20.32	133.9 ± 22.36	139.9 ± 18.1	0.8957
**PLT (×10^9^/L)**	179.91 ± 84.16	170.30 ± 88.10	185.24 ± 81.71	0.0845
**ALT (U/L)**	58.38 ± 40.62	65.28 ± 44.99	54.54 ± 37.60	0.0712
**AST (U/L)**	84.89 ± 62.44	91.17 ± 63.80	81.40 ± 61.62	0.1403
**ALB (g/L)**	40.10 ± 4.40	39.30 ± 4.55	40.54 ± 4.26	0.0629
**TBIL (μmol/L)**	16.44 ± 7.61	17.73 ± 8.49	15.72 ± 6.99	0.0517
**AFP (< 1,000 ng/mL: > 1,000 ng/mL)**	138:104	54:32	86:70	0.9048
**PT (s)**	12.78 ± 2.08	11.84 ± 2.05	13.44 ± 2.09	0.0515
**APPT (s)**	27.85 ± 3.85	28.22 ± 3.69	27.70 ± 3.94	0.2777
**HBV-DNA (log_10_ IU/mL)**	4.85 ± 1.86	5.10 ± 1.58	4.88 ± 1.92	0.09214
**TNM stage (I:II:III:IV)**	43:22:145:32	20:12:46:8	23:10:99:24	0.0737

Abbreviations: AFP, α-fetoprotein; ALB, serum albumin; ALT, alanine aminotransferase; APTT, activated partial thromboplastin time; AST, aspartate aminotransferase; HBeAg, hepatitis B e antigen; HBG, hemoglobin; HBV, hepatitis B virus; PLT, platelets; PT, prothrombin time; TACE, transarterialchemoembolization; TBIL, total bilirubin; UICC TNM, International Union Against Cancer Tumor-Node-Metastasis; WBC, white blood cells.

### Propensity score matching

Propensity score matching generated 81 pairs of patients for the various combinations of chemotherapeutic agents with mixed lipiodol, which could cause complications directly affecting all parameters (Table 2). Table 2 shows the characteristics of participants receiving or not receiving parecoxib in both the pre-match and post-match samples. For the covariates 5-fluorouracil deoxyriboside (FUDR) and Platinum, *t* tests (for continuous variables) andchi-square analyses (for discrete variables) showed significant differences between patient samples receiving parecoxib and those treated without parecoxib before matching. After matching, differences among covariates for those receiving or not receiving parecoxib were reduced. None of variables had significant *P* values in *t*-tests or chi-square tests.

**Table 2:** Covariate comparison between groups before and after propensity score matching

	Pre-Matching				Post-Matching			
	Parecoxib	Non-Parecoxib	Statistics	*P*-Value	Parecoxib	Non-Parecoxib	Statistics	*P*-Value
**Number (%)**	86 (35.5)	156 (64.5)			81 (50.0)	81 (50.0)		
**Age (years)**	52.79 ± 13.45	52.88 ± 11.15	*t* = 0.06	0.959	52.54 ± 13.71	52.89 ± 11.82	*t* = 0.09	0.864
**Iodized oil (mL)**	13.22 ± 8.79	11.82 ± 8.09	*t* = 1.22	0.225	12.90 ± 8.85	13.28 ± 8.76	*t* = 0.28	0.783
**EADM (%)**				0.973				1
**Yes**	85 (98.8)	153 (98.1)	Fisher		80 (98.8)	80 (98.8)	Fisher	
**No**	1 (1.2)	3 (1.9)			1 (1.2)	1 (1.2)		
**MMC(%)**								
**Yes**	51 (59.3)	85 (54.5)	χ^2^ = 0.34	0.557	46 (56.8)	42 (51.8)	χ^2^ = 0.22	0.636
**No**	35 (40.7)	71 (45.5)			35 (43.2)	39 (48.2)		
**FUDR (%)**								
**Yes**	22 (25.6)	23 (14.7)	χ^2^ = 3.62	0.049	17 (21.0)	16 (19.8)	χ^2^ = 0.00	1
**No**	64 (74.4)	133 (85.3)			64 (79.0)	65 (80.2)		
**Platinum (%)**								
**Yes**	53 (61.6)	121 (77.6)	χ^2^ = 6.20	0.013	53 (65.4)	56 (69.1)	χ^2^ = 0.35	0.738
**No**	33 (38.4)	35 (22.4)			28 (34.6)	25 (30.9)		
**Pain score (D_x_)**	0.72 ± 1.72	0.96 ± 1.69	*t* = 1.05	0.098	0.69 ± 1.66	0.83 ± 1.58	*t* = 1.03	0.595
**Vomiting (D_x_)**	0.00 ± 0.00	0.02 ± 0.14	*t* = 1.74	0.198	0.00 ± 0.00	0.01 ± 0.11	*t* = 1.00	0.323
**Temperature (D_x_,°C)**	36.74 ± 0.27	36.71 ± 0.31	*t* = 0.91	0.366	36.73 ± 0.27	36.72 ± 0.32	*t* = 0.67	0.729

### Effects of parecoxib on pain scores and body temperature

**Figure 1:**
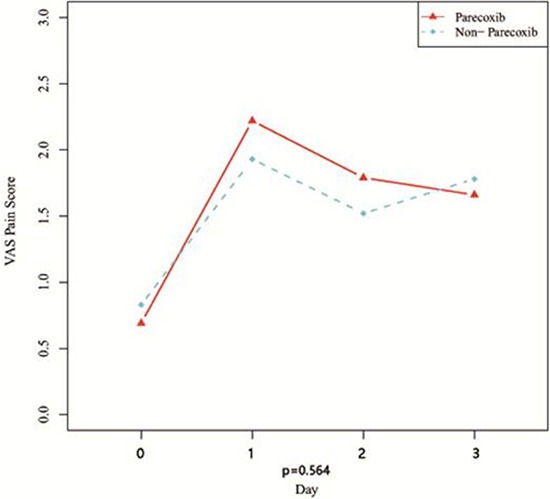
Curves of VAS pain score after TACE

**Figure 2:**
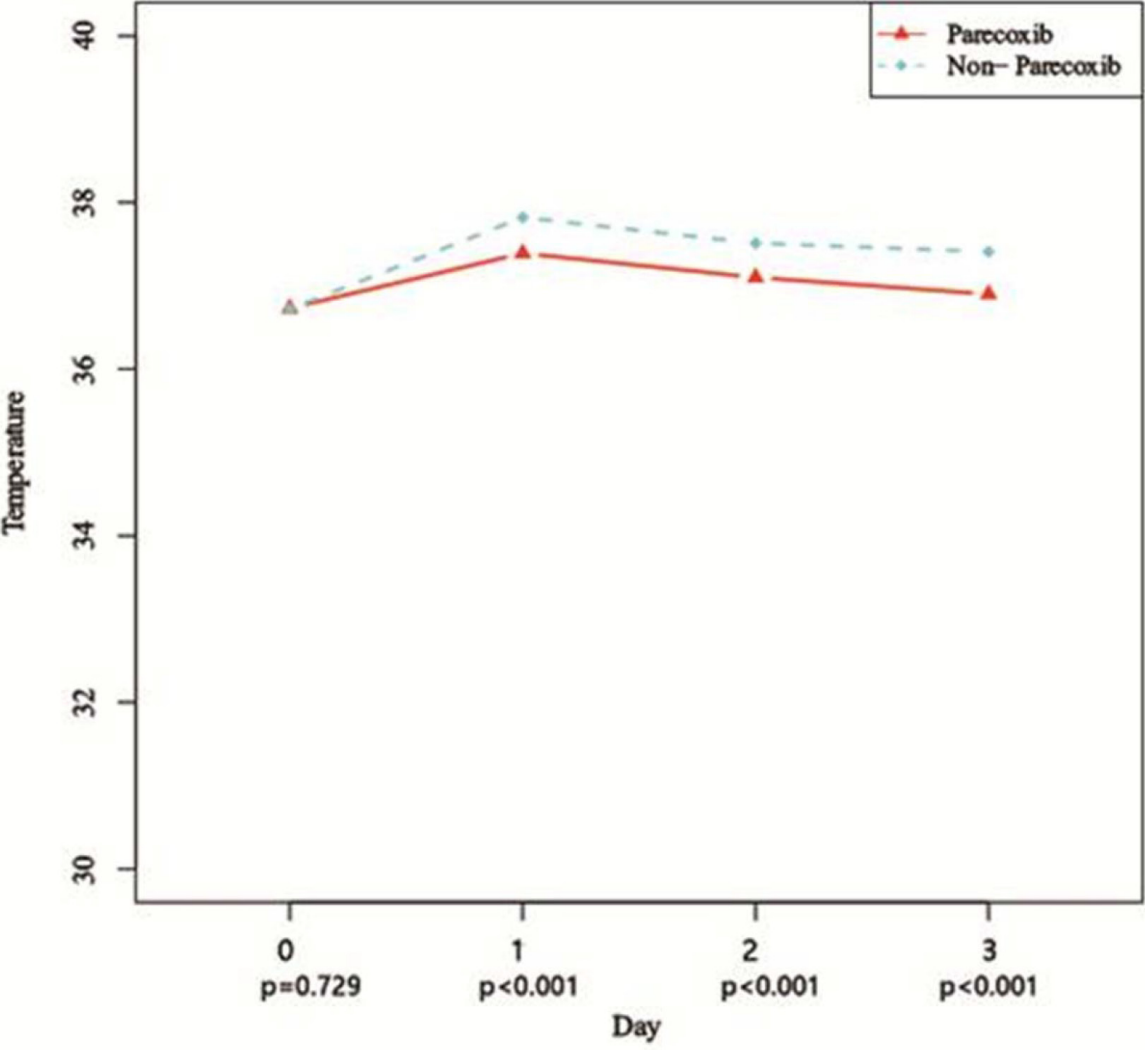
Curves of body temperatures after TACE

Comparing VAS pain scores on D_x_ after TACE, revealed the scores to be significantly higher than the preoperative scores in both groups (*P* < 0.01). On the other hand, there were no significant between-group differences (*P* > 0.05) in VAS scores at any time point (Table 3). Similarly, preoperative body temperatures were significantly lower than the temperatures after TACE (*P* < 0.01). But compared to the control group, body temperatures in the Parecoxib group were lower on D_1_, D_2_, and D_3_(*P* < 0.001) after TACE (Figure 1 and Figure 2).

**Table 3:** Comparison of related complications, physical activities and tumor control after TACE

	Parecoxib (*n* = 81)	Non-Parecoxib (*n* = 81)	*P*-Value
**VAS pain score**			
D0	0.69 ± 1.66	0.83 ± 1.58	0.564
D1	2.22 ± 2.31	1.93 ± 2.07	
D2	1.79 ± 2.07	1.52 ± 1.90	
D3	1.66 ± 1.98	1.78 ± 1.07	
**Temperature**			
D0	36.73 ± 0.27	36.72 ± 0.32	0.729
D1	37.39 ± 0.69	37.82 ± 0.73	**< 0.001**
D2	37.10 ± 0.52	37.51 ± 0.74	**< 0.001**
D3	36.90 ± 0.33	37.41 ± 0.65	**< 0.001**
**Vomiting**			
D0	0.00 ± 0.00	0.01 ± 0.11	0.923
D1	0.34 ± 0.75	0.27 ± 0.84	
D2	0.04 ± 0.25	0.11 ± 0.71	
D3	0.09 ± 0.34	0.15 ± 0.63	
**ALT(U/L)**	86.97 ± 147.68	118.08 ± 197.05	0.265
**AST(U/L)**	154.79 ± 228.48	158.38 ± 213.19	0.914
**CRP (mg/L)**	41.30 ± 42.61	44.85 ± 43.84	0.593
**ALB (g/L)**	33.6 ± 6.84	34.1± 4.58	0.124
**TBIL (μmol/L)**	24.98 ± 15.46	27.24 ±10.73	0.097
**Physical Activity**			**0.028**
Low	16 (19.75%)	29 (35.80%)	
Moderate	65 (80.24%)	52 (64.20%)	
High	0	0	
**Hospital stay (days)**	7.21 ± 2.05	7.92 ± 2.64	**0.041**
**Tumor control**(CR:PR:SD:PD)	12:45:18:6	9:52:13:7	0.554

### Postoperative vomiting and liver inflammation

The side effects related to parecoxib in study group were not significant. Vomiting in patients was worse after TACE than before the operation (*P* < 0.01). As shown in Table 3, there were no statistically significant differences between the groups (*P* > 0.05)with respect to vomiting on D_0_, D_1_, D_2_, orD_3_. Liver function abnormalities were observed early after TACE in both groups, and the distributions of ALT, AST, ALB and TBIL in those patients were similar in the two groups (*P* > 0.05).

### Effect of parecoxib on physical activity, hospital stay and short-term outcome

After TACE, patients in the parecoxib group exhibited greater physical activity (*P* = 0.028) and had shorter hospital stays (*P* = 0.041) than patients in the non-parecoxib group (Table 3). However, modified RECIST performed to assess short-term outcome revealed no difference between the distributions in the two groups (Table 3).

## DISCUSSION

As a minimally invasive treatment for liver cancer, TACE has the advantages of reduced trauma, low cost, and a short hospital stay. After TACE, however, tumor necrosis causes inflammation, with fever being the most common symptom [10, 11]. Our study found that the COX-2 inhibitor parecoxib sodium effectively reduces postoperative fever. The average body temperatures of the parecoxib group were lower than those of the control group at several time points after TACE. In addition, the average hospital stay was shorter for the parecoxib group than the control group. This may be due to parecoxib's ability to control body temperature and so reduce patient discomfort, thereby accelerating postoperative recovery, which was also reflected by the patients' greater physical activity.

Pain management is an important part of cancer treatment that is receiving increased attention. The VAS scores of patients were significantly increased after TACE, due to pain caused by hypoxia of local tissue, tumor necrosis, swelling of the capsule or ectopic embolization [12–14]. Analysis based on the volumes of iodized oil used, which plays a major role in TACE, showed that the postoperative pain scores for patients who received more than 10 ml of iodized oil were significantly higher than those for patients receiving less than 10 ml of iodized oil. These results suggest that early postoperative pain is closely related to the amount of iodized oil used and that a postoperative analgesia scheme should be developed in accordance with the amount of intraoperative oil used.

Although the efficacy of parecoxib sodium for postoperative analgesia has been reported, our study did not confirm its value in pain control after TACE. Specifically, we detected no significant difference between the parecoxib and control groups with respect to postoperative pain scores at several time points. There are several possible reasons for this negative result. First, compared to procedures used in gynecology, orthopedics, and general surgery, TACE is a minimally invasive surgical intervention, and postoperative pain is not as severe. Moreover, in this study, the average VAS pain score at each time point was < 4, which does not meet the criteria for moderate to severe pain, making the analgesic effect of parecoxib sodium undetectable. In addition, previous studies have primarily considered parecoxib sodium toact in concert with other potent analgesic drugs [15–17]. Used alone, parecoxib sodium may not have significant effects.

Parecoxib sodium inhibits inflammation *in vivo* by reducing prostaglandin synthesis, thereby suppressing leukocyte aggregation and reducing the formation of bradykinin [18]. After TACE, the effects of chemotherapy drugs and local liver ischemia due to hepatic artery embolization can lead to liver inflammation and damage [19, 20]. Our study found that postoperative application of parecoxib sodium had no significant effect on the levels of inflammatory markers, including ALT, AST, ALB, TBIL and CRP. During its conversion to valdecoxib, parecoxib sodium is degraded and metabolized in the liver. In theory, this process could increase the burden on the liver. It was found, however, that this process has no significant effect on liver function in healthy individuals. It can thus be concluded that parecoxib sodium metabolism does not increase the burden on the liver *in vivo*, and that its inflammation-inhibiting effects may be masked by postoperative application of various hepatoprotective drugs routinely used after TACE [21]. Vomiting is another common adverse postoperative reaction of TACE. Our study confirmed that parecoxib sodium does not exacerbate vomiting, and it remains to be determined whether prophylactic application of antiemetic medication is necessary to optimize the postoperative quality of life in the short term.

COX-2 is overexpressed in HCC and is thought to contribute to hepatocarcinogenesis [22]. Consistent with that idea, inhibition of COX-2 reportedly enhances chemotherapeutic efficacy in preclinical research and cancer clinical trials [23, 24]. Although there was no difference between the effect of TACE on tumors in the parecoxib and control groups in our study, we can draw no definite conclusion yet, since this is a short-term medication.

In summary, postoperative application of parecoxib sodium after TACE effectively reduces fever and promotes patients' physical recovery, thereby shortening their hospital stay. The effects of parecoxib sodium on pain and liver inflammation are less obvious, and the effects of combining parecoxib sodium with other analgesics for pain control and inflammation inhibition should be tested in a future study. Moreover, because this was a non-randomized controlled study, assessing parecoxib and its anti-cancer effect in HCC will require further investigation.

## PATIENTS AND METHODS

### Patients and inclusion criteria

This prospective study was approved by the Institutional Review Board (IRB) at the Sun Yat-sen University Cancer Center, and allmethods were carried out in accordance with the approved guidelines. (ClinicalTrials.gov ID: NCT02552745, Date of registration: September 11, 2015).

The patients included in the study were diagnosed with HCC based on the criteria established by the European Association for the Study of the Liver [25], had a previous history of hepatitis B or positivity for hepatitis B surface antigen (HBsAg), had received no treatment for liver cancer prior to participating in the study, had a Karnofsky Performance Status (KPS) score ≥ 70, were between 18 and 65 years of age, and were Child-Pugh class A or B (class B patients had scores no greater than 7). In addition, the baseline laboratory tests of the included patients had to meet the following criteria: white blood cell counts (WBCs) ≥ 1.5 × 10^9^/L, platelets ≥ 50 × 10^9^/L, hemoglobin ≥ 80 g/L, serum aspartate transaminase (AST) and alanine transaminase (ALT) ≤ 2 × the upper limit of normal (ULN), serum creatinine ≤ 1.5 × ULN, an international normalized ratio (INR) < 1.5 or prothrombin time < the ULN + 4 seconds, albumin ≥ 30 g/L, and total bilirubin ≤ 34 mmol/L.

Excluded were patients who had iodine allergies, severe heart and lung diseases, significant fever or pain, or continuous use of anti-inflammatory drugs within the past three months.

### Patients grouping

The administration of parecoxib sodium was based on the clinical experience of the physicians and surgeons. Patients were divided into a study group (receiving postoperative parecoxib sodium) and a control group (without parecoxib sodium). To minimize bias, propensity score matching was performed, and 81 pairs of propensity score-matched patients were involved in the final analyses.

### TACE treatment procedures and postoperative management

To perform TACE, the tip of the catheter was placed into the tumor-feeding artery, and one or several chemotherapy drugs, mixed with iodized oil, slowly injected into the tumor-feeding artery. The chemotherapeutic drugs included epirubicin (50 mg per application) and/or mitomycin C (6 mg per application), carboplatin (300 mg per application), lobaplatin (50 mg per application), and floxuridine (500 mg per application). The particular chemotherapeutic drugs and the volume of iodized oil used for each patient were determined in accordance with tumor conditions, and the TACE conditions were recorded. After surgery, the study group received 40 mg parecoxib sodium dissolved in 10 ml of saline via intravenous injection. This administration protocol was conducted once every 12 hours for three days. The control group received no parecoxib sodium. Instead, they received symptomatic treatment with tramadol and acetaminophen when necessary.

### Observation indicators and detection methods

The variables D_x_, D_0_, D_1_, D_2_, and D_3_ represent the values before TACE, 2 hours postoperative, and the first, second, and third postoperative days, respectively. The highest visual analogue scale (VAS) pain scores of the day, the vomiting grade, and the maximum daily temperature at each of the time points were recorded. Also collected were the results of routine biochemistry examinations using venous blood during re-examinations on D_2_ and D_3_, as well as the length of the hospital stay. The VAS pain score was determined using a scale of 0–10, with 0 being painless and 10 being unbearable pain. The vomiting grading criteria were as follows: grade 0, no vomiting; grade I, mild vomiting (one to two times/day); grade II, moderate vomiting (three to five times/day); and grade III, severe vomiting (greater than five times/day). From the routine biochemical test results conducted as part of the postoperative re-examination, changes in alanine aminotranferease(ALT), aspartate aminotransferase (AST), serum albumin (ALB), total bilirubin (TBIL) and C-reactive protein (CRP) levels were used to assess postoperative liver function changes. Physical activity levels were evaluated using the self-administered long form of the International Physical Activity Questionnaire (IPAQ), and patients were categorized into 3 levels of physical activity: low, moderate, and high [26]. Tumor characteristics and TNM stage (Union for International Cancer Control, UICC, 7th version) were evaluated through imaging and/or intra-operative observation. Tumor response rate was evaluated using Modified Response Evaluation Criteria in Solid Tumors (mRECIST) one month after TACE [27].

### Statistical methods

Data were analyzed using SAS 9.1.software (SAS Institute, Cary, NC). The statistical analysis was conducted using *t* tests and repeated-measures analysis of variance. The results are expressed as the mean ± the standard deviation. Values of *P* < 0.05 was considered statistically significant.
